# Gene Flow Patterns among *Aedes aegypti* (Diptera: Culicidae) Populations in Sri Lanka

**DOI:** 10.3390/insects11030169

**Published:** 2020-03-06

**Authors:** H.S.D. Fernando, Menaka Hapugoda, Rushika Perera, William C. Black IV, B.G.D.N.K. De Silva

**Affiliations:** 1 Center for Biotechnology, Department of Zoology, Faculty of Applied Sciences, University of Sri Jayewardenepura, Nugegoda 10250, Sri Lanka; sachini@sci.sjp.ac.lk; 2 Molecular Medicine Unit, Faculty of Medicine, University of Kelaniya, Kelaniya 11010, Sri Lanka; menakaha@yahoo.com; 3 Department of Microbiology, Immunology and Pathology, Colorado State University, Fort Collins, CO 80523, USA; Rushika.Perera@colostate.edu (R.P.);

**Keywords:** *Aedes aegypti*, population structure, gene flow patterns, Sri Lanka

## Abstract

In Sri Lanka, dengue is the most serious arboviral disease. Recent increases in dengue cases suggest a higher infection rate and spread of the disease to new areas. The present study explores gene flow patterns of *Ae. aegypti*, the main vector of dengue disease, among 10 collection sites including major ports and inland cities using variations at 11 microsatellite loci. Discriminant analysis of principal components (DAPC) and *k*-means clustering estimated eight genetic clusters. Analysis of Molecular Variance (AMOVA) estimated equal variances among cities and among collections in Colombo, Sri Lanka. Significant evidence, although weak, was detected for isolation by distance. Analysis of gene flow rates and directions using MIGRATE-n indicated that populations throughout the island served as a source of immigrants for Colombo with abundant gene flow among major commercial cities in Sri Lanka, which appear to receive migrant mosquitoes from throughout Sri Lanka. The observed patterns probably arise through human movement of *Ae. aegypti* during commerce from throughout Sri Lanka into Colombo increasing the risk of spread. The patterns uncovered in this study are significant for global health as Sri Lanka is situated along a key international shipping route.

## 1. Introduction

Viruses transmitted by mosquitoes are a primary health concern driven by multiple factors leading to the loss of human life due to the increased incidence of mosquito-borne diseases globally [1]. Dengue fever (DF) is an acute mosquito-borne viral disease transmitted principally by *Aedes aegypti,* which has been on the rise over the past 50 years with a 30-fold increase in global incidence [1,2]. *Aedes aegypti* is an invasive mosquito species originated in Africa and has colonized tropical and subtropical regions of the world during the last 400–500 years with the help of human movement and trade [3].

Sri Lanka is a tropical island situated in the Indian Ocean. The island is situated along a key international shipping route between the Malacca Straits and the Suez Canal. Approximately 36,000 ships, including 4500 oil tankers, use the route annually [4]. Sri Lanka is also a major tourist attraction site with hundreds of tourists from all over the world visiting the country throughout the year.

In Sri Lanka, DF is considered a serious arboviral disease with progressively larger epidemics over regular periods of time [5]. Although deaths due to dengue hemorrhagic fever (DHF) and dengue shock syndrome (DSS) have been decreasing in Sri Lanka due to better clinical management, reports of an increase in the frequency and magnitude of DF cases suggest that the infection rate is still high [6]. According to the health ministry of Sri Lanka, 2017 marked the highest number of DF cases reported, with a record-breaking 185,690 DF cases and 215 deaths [7]. This is 4.7-folds higher than the average number of cases for the same period between 2010 and 2016 [7]. The 2017 DF outbreak occurred after heavy rains and flooding and affected 15 out of 25 districts in Sri Lanka [7]. Based on sentinel site surveillance over seven years, the expected peak months of May to July coincide with the south western monsoon that commences in late April [8]. During the year 2019, 104,500 cases were reported and approximately 43% of DF cases were reported from the Western Province. The most affected area was the Colombo District with 20,718 reported cases [7]. Four known serotypes have been co-circulating in Sri Lanka for more than 30 years [8]. Heavy monsoon rains, public failure to clear rain-soaked garbage, standing water pools and other potential breeding grounds for mosquito larvae contribute to the higher number of DF cases reported in urban and suburban areas.

While DF is a significant public health threat in Sri Lanka, these patterns would have little significance to global health if not for the fact that Sri Lanka is situated along a key international shipping route. Thus, Sri Lanka may be playing a major role in the trafficking of DENVs, its vector *Ae. aegypti* and infected humans from Southeast Asia to the Middle East and Africa. A recent study assessing the genetic relationships of the Sri Lankan population of *Ae. aegypti* relative to other global collections revealed Sri Lankan mosquitoes cluster with other Asian collections [3]. Among Asia/Pacific collections, the Sri Lanka collection was most similar to individuals from Pakistan and Jeddah, Saudi Arabia. Recent analyses using whole-genome sequencing [9] have also revealed strong similarity among mosquitoes collected from Jaffna and Batticaloa in Sri Lanka and from independent sites in Yucatán state in Mexico [9].

Knowledge of dispersal of a species gives insight into the gene flow levels and the adaptability of that particular species to a new area. Thus, understanding the genetic structure of *Ae. aegypti* within Sri Lanka gives an understanding regarding the population movements, the flow of resistant genes and new epidemics that could occur.

**Figure 1:**
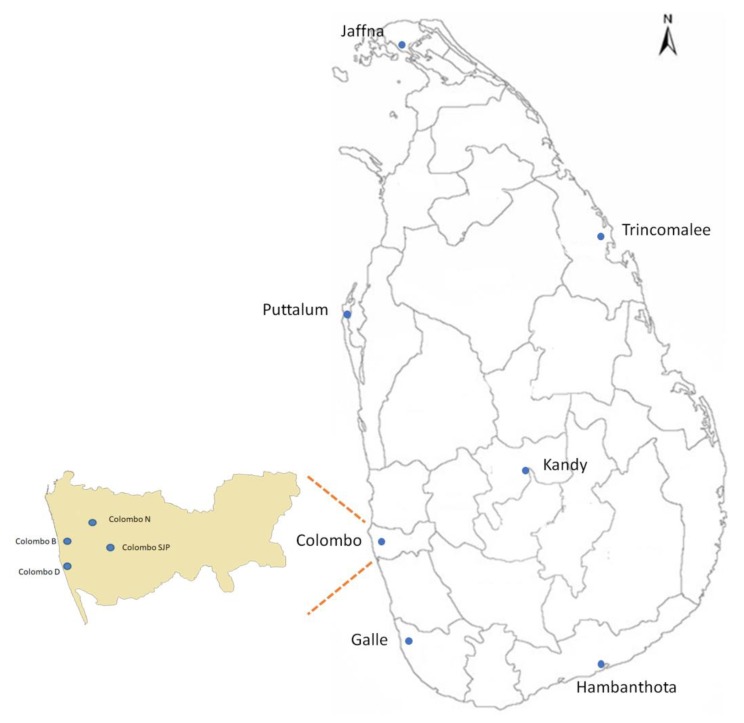
Location of *Ae. aegypti* collection sites in Sri Lanka. Blue dots indicate the sampling sites and light gray lines indicate the district boundaries on the map.

We obtained collections from 10 sites in Sri Lanka, including four collections from various locations within Colombo (B, D, N and J) and single collections from Jaffna, Galle, Hambanthota, Puttalum, Trincomalee and Kandy (Figure 1; Supplementary Tables S1 and S2). Colombo, the commercial capital of Sri Lanka, is the largest city in the country, with the highest population density. The Port of Colombo (Figure 1) ranks among the top 35 ports in the world and has one of the biggest artificial harbors [10]. It is an important terminal in Asia due to its strategic location in the Indian Ocean. It is a major international container trans-shipment hub, handling most of the country’s foreign trade. Hambantota (Figure 1) is a harbor close to international shipping routes on the southern tip of Sri Lanka [10], which has been opened recently in 2010 to provide facilities for port-related industries and services not provided by Colombo. Trincomalee (Figure 1) is a natural deep-water harbor in northeast Sri Lanka and is the second-largest natural harbor in the world [10]. It is accessible to all types of vessels in all weather conditions. It is approximately 10 times larger than the Port of Colombo. Galle (Figure 1) is the capital city, a regional port and a popular tourist attraction in southern Sri Lanka. It is a World Heritage Site and may be involved in the movement of mosquitoes and DENVs since many tourists visiting Colombo take the train or a bus to Galle. *Aedes aegypti* were also collected in three inland sites. Jaffna (Figure 1) is the capital city of the Northern Province of Sri Lanka. Prior to the civil war, it was Sri Lanka’s second most populated city (the first being Colombo). Since the end of the war in 2009, refugees and internally displaced people have returned to their homes and government and private sector reconstruction has begun. This immigration may contribute to the movement of mosquitoes and DENVs. Kandy is a well-known tourist attraction site as well (Figure 1). Puttalam (Figure 1) is an industrial center known for energy production, salt, coconut production and fishing.

The current study analyzed the gene flow patterns and the population genetic structure of *Ae. aegypti* in Sri Lanka. The main aim of the present study was to analyze the genetic structure and gene flow of the populations of *Ae. aegypti* collected from four major ports cities and three inland study sites in Sri Lanka.

## 2. Materials and Methods

### 2.1. Mosquito Collection and Genomic DNA Isolation

Adults and larvae of *Ae. aegypti* were collected from 2013 to 2015 from seven districts (Figure 1). Sample details appear in Supplementary Tables S1 and S2. Samples were collected using BG-sentinel adult mosquito traps and ovitraps. Collected larvae were reared to adults and killed by freezing. All adults were morphologically identified to species using standard taxonomic keys [11]. Identified adults were preserved by desiccation with silica gel. For DNA extraction, a maximum of five individuals per trap were selected to avoid over-sampling of siblings. DNA was extracted from individual adults using a phenol/chloroform method [12]. Extracted DNA was stored in 100 µL TE buffer (10 mM Tris, 0.1 mM EDTA, pH 7.5–8.0).

### 2.2. Microsatellite Genotyping

Eleven previously published nuclear microsatellite loci AT1, AG7, AC1, AG5, AG4, AC2, AG2, AC5 [13], B07, H08 and A10 [14] were amplified in 204 individuals in 10 collection sites using primers labeled fluorescently with the ROX set of dyes (i.e., 6-FAM (Blue), HEX (Green)). Microsatellite PCR was completed in a volume of 25 µL containing 5 ng of genomic DNA, 0.4 µM of forward and reverse primers, 2.0 mM MgCl_2_, 0.1 mM dNTPs, 1 × PCR amplification buffer and 0.4 units of *Taq* polymerase (Promega, Fitchburg, WI, USA). The polymerase chain reaction (PCR) was carried out in an Eppendorf^®^ Thermo-cycler in 25 µL reaction volumes. Amplification conditions consisted of initial denaturation at 95 °C for 15 min followed by 35 cycles of 94 °C for 30 s, 55 °C for 90 s and 72 °C for 90 s and a final extension step at 72 °C for 1 min. PCR products were sent to Macrogen Inc. Korea for fragment analysis. The results obtained were genotyped using GeneMapper™ Software 5 with GeneScan™ 1200 LIZ™ dye Size Standard (Applied Biosystems, Foster City, CA, USA). All loci were polymorphic.

### 2.3. Population Structure Analyses

The observed and expected heterozygosities, (H_o_ and H_e_), allelic richness and the Garza–Williamson (GW) Index [15] were estimated at the 11 loci in each population using ARLEQUIN, version 3.5.2.2 [16]. This software was also used to calculate the significance of deviations from Hardy–Weinberg (HW) equilibrium for each locus in each collection (10,000 Markov chains) and to test for the presence of linkage disequilibrium (LD) between alleles at all possible pairs of loci in each collection. Null allele frequencies were calculated using CERVUS [17]. The alleles which recorded a null allele frequency over 0.15 were removed from further analysis. Analysis of Molecular Variance (AMOVA) was used to estimate the amount of gene flow within populations, among populations within Colombo and among cities. AMOVA and Isolation by distance were evaluated using ARLEQUIN version 3.5.2.2 [16]. 

The population structure was analyzed by a principal component analysis (PCA) and discriminant analysis of principal components (DAPC) using adegenet 2.1.1 [18] for R version 3.2.1 [19,20]. In DAPC analysis, the raw data is first transformed through a PCA and then a discriminant analysis (DA) is performed on the retained principal components (PCs). All individuals are assigned to the population for which they have the highest probability and any individual assigned to a population that is not their true population of origin are misclassified individuals. The find.clusters function was used to detect the number of clusters in the population with the use of *k*-means clustering. The lowest Bayesian Information Criterion (BIC) was used to infer the ideal value of K. The first two principal components of the DAPC were plotted to evaluate the relationship among clusters. A cross-validation function (Xval.dapc) was used to confirm the correct number of principal components (PC) to be retained.

Populations, which have experienced a recent reduction of their effective population size, exhibit a correlative reduction in allele numbers and heterozygosities at polymorphic loci. Data were analyzed for evidence of a bottleneck using the program (BOTTLENECK version 1.2.02) [21]. To determine whether a population exhibits a significant number of loci with heterozygosity excess, two tests, namely a “Wilcoxon sign-rank test” [22] and a descriptor of the allele frequency distribution (“mode-shift” indicator), were applied. The program provides results under three mutation models; the infinite allele model (IAM), the stepwise mutation model (SMM) and the two-phase mutation model (TPM).In practice, microsatellite evolution varies among loci and falls in a range boarded by the two extreme models of mutation IAM and SMM and the TPM model has been considered to better describe microsatellite data. We used a TPM of mutation with a 10% infinite allele model and a 90% SSM with a 15% variance for 1000 iterations. Significance was assessed using Wilcoxon’s signed-rank test. The second method was based on allele frequency distribution and the program tests whether the allele frequency distribution is approximately L-shaped, which is expected under mutation-drift equilibrium. A shift in the allele frequency would indicate a recent bottleneck. The Garza–Williamson Index (calculated in ARLEQUIN version 3.5.2.2 [16]) discriminates bottlenecked populations from stable populations [23].

Migrate-n was used to estimate two parameters: the effective migration rate (4NeM) and θ = 4Neµ. Two replicate maximum likelihood runs were made in MIGRATE-n 3.5.1 [24]. As recommended in the instructions for MIGRATE-n3.5.1., we assumed that a Brownian Motion Model with 20 short chains with 500 recorded steps, 100 increments, 50,000 sampled genealogies and the number of discarded tree per chain was 10,000. Two long chains were used with 5000 recorded steps, 100 increments and 500,000 sampled genealogies. To test for convergence, Pearson correlation coefficients were calculated between 4N_e_m values estimated in the first and second replicates to test for the consistency of estimates from the two replicate runs. The same was done with θ. The immigration parameter M (not m) among populations and θ within populations were estimated using maximum likelihood in Migrate-n 3.5.1 [24]. The effective migration rate 4NeM between a donor population and a recipient population was calculated by multiplying the immigration parameter M (MIGRATE-n output) by the recipient population θ. When 4NeM < 1, the migration rate between the two populations was not sufficient to neutralize the effects of genetic drift, enabling allele frequencies to diverge between populations. When 4NeM > 1, there was sufficient migration to maintain homogeneity between donor and recipient populations. We classified each of the 10 collection sites as a “source” if it contributed emigrants to other collection sites with 4NeM > 1 or as a “sink” if it received immigrants from other populations at a rate 4NeM > 1.

## 3. Results

**Figure 2:**
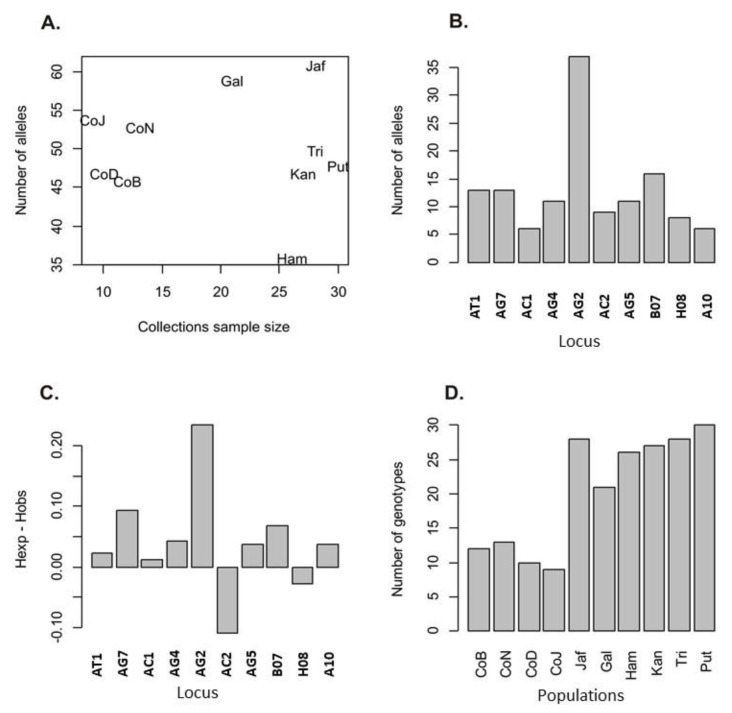
(**A**) The relationship between the number of alleles recorded in *Aedes aegypti* and collection sample size. (**B**) The number of alleles per locus. (**C**) The differences between expected and observed heterozygosity and sample size in each collection. (**D**) Sample size in each collection.

There was no obvious correlation between sample sizes and the numbers of alleles detected in a collection (Figure 2A). The number of alleles detected per locus varied between five for loci AC1 and A10 to 36 for locus AG2 (Figure 2B). Heterozygosity expected (H_e_) exceeded the observed heterozygosity (H_o_) in eight of the 10 loci (Figure 2C). The greatest excess in H_e_ was observed in locus AG2 that also had the largest number of alleles. Null alleles were frequent at locus AC5 and it was therefore excluded from further analysis. Linkage disequilibrium was not detected among the loci studied.

We found that 91% of the variance in allele frequencies arose within populations while 4.8% arose among cities and 4.1% among collections within Colombo (Table 1). This pattern within and among cities could be interpreted as arising from genetic drift but only two populations exhibited evidence of a bottleneck. These were Colombo J and Hambanthota. The mode shifts for these two populations are shown in Supplementary Figure S1. The GW statistic is small in populations having been through a bottleneck and close to one in stationary populations. Colombo J and Hambanthota and Trincomalee had the smallest average Garza Williamson Indices (Table 2). The sign test for Hambanthota with the infinite allele model (IAM) had no loci with heterozygote deficiency and 10 loci with heterozygote excess (P = 0.0015) but not for the stepwise mutation model (SMM) (P = 0.121). The sign test for Colombo J was not significant for IAM or SMM. The sign test for Trincomalee with the infinite allele model (IAM) had no loci with heterozygote deficiency and 10 loci with heterozygote excess (P = 0.0018) but not for the stepwise mutation model (SMM) (P = 0.121).

**Figure 3:**
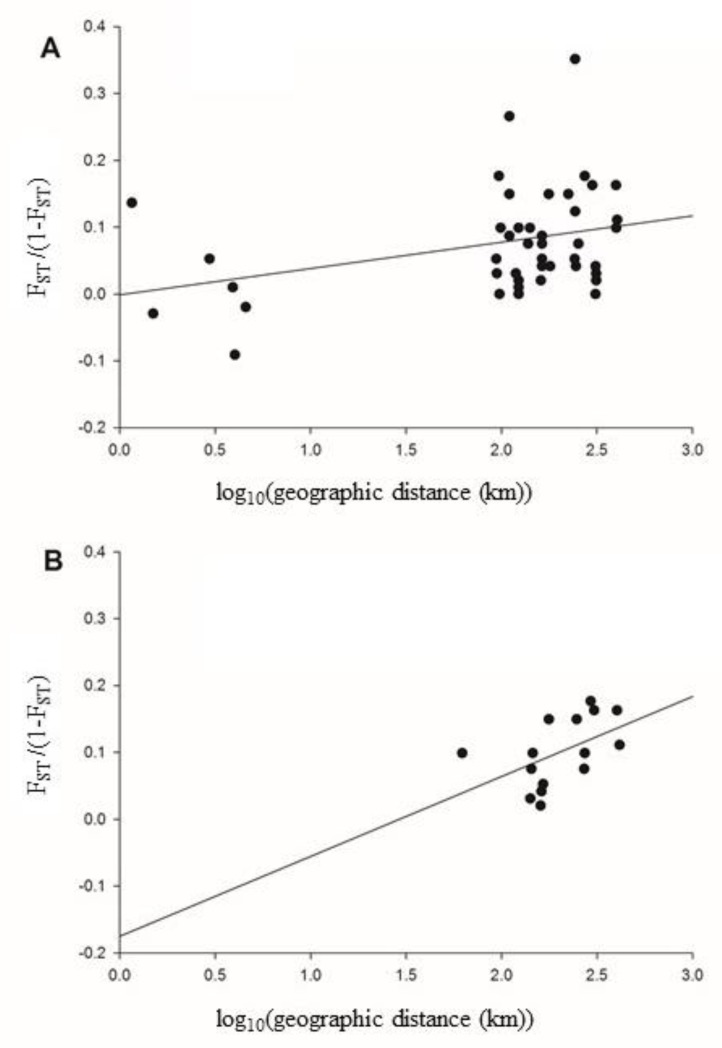
Scatter plot and regression line of genetic and geographic distance for all Sri Lankan *Ae. aegypti* populations. ARLEQUIN was used to test the correlation between the linear genetic distance (F_ST_/(1-F_ST_)) and the log_10_ (geographic distance) using a Mantel test. A positive correlation was observed between the geographic distance and the genetic distance (r^2^ = 0.11, p = 0.0001) when including all populations (**A**). This revealed that *F_ST_* increased with increasing geographic distance thus there is some, albeit weak, evidence for isolation by distance among collections. A positive correlation was observed between the geographic distance and the genetic distance (r^2^ = 0.50, p = 0.0001) when excluding those populations that were close to one another (**B**).

ARLEQUIN was used to test the correlation between the linear genetic distance (F_ST_/(1-F_ST_)) and the natural log (geographic distance) using a Mantel test (Figure 3). A positive correlation was observed between the geographic distance and the genetic distance (r^2^ = 0.11, p = 0.0001) (Figure 3A). This revealed that *F_ST_* increased with increasing geographic distance, thus there is a significant but weak correlation between genetic and geographic distance within Sri Lanka. A positive correlation was observed between the geographic distance and the genetic distance (r^2^ = 0.50, p = 0.0001) when excluding the Colombo populations that were close to one another (Figure 3B).

**Figure 4:**
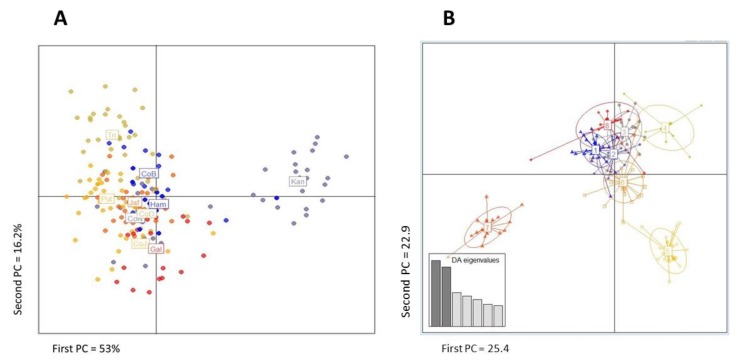
(**A**) Principal component analysis (PCA) of the original data in ten collections from Sri Lanka. Each dot corresponds to an individual mosquito. The amount of variance explained by each PC is indicated on each axis. The first two principal components account for 53% (X-axis) and 16.2% (Y-axis) of the total variance. (**B**) Discriminant Analysis of principal components (DAPC) displayed as eight genetic clusters. The first and second principal components account for 25.4 + 22.9 = 48.3 of the total variation. Visual clusters of points of the same color in Figure 3A correspond roughly to those in Figure 3B.

**Figure 5:**
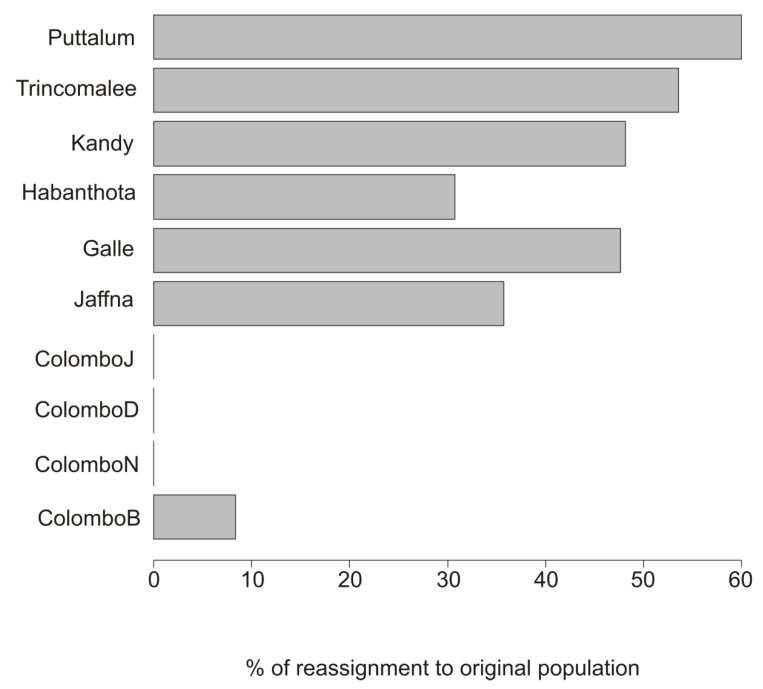
Misclassification of *Ae. aegypti* individuals in populations from Sri Lanka. Bars indicate the percentage of individuals that are reassigned to their original collection site.

The PCA plot (Figure 4A) explains 69% of the overall variation. However, only the Kandy population forms a distinct cluster. Adegenet 2.1.1 identified the numbers and sizes of genetic clusters with the lowest BIC and eight genetic clusters were identified (Supplementary Figure S2). DAPC and *k*-means clustering show that Clusters 1, 3, 6 and 8 contained individuals from many (8–10) collection sites. In contrast, Clusters 2 and 4 contained primarily mosquitoes from Trincomalee while Cluster 7 consisted almost exclusively of Puttalum individuals. Cluster 5 consists primarily of individuals from Galle, Hambanthota and Kandy (Figure 4B). This pattern is consistent with the misclassification patterns in Figure 5, wherein between 30% and 60% of individuals were reassigned to their original collection site whereas none of the mosquitoes collected from Colombo J, D, N were reassigned to their original collection and only 9% of collected individuals were reassigned to Colombo B. This suggests that there was some mixing of individuals in populations outside Colombo and complete mixing of individuals in Colombo. 

**Figure 6:**
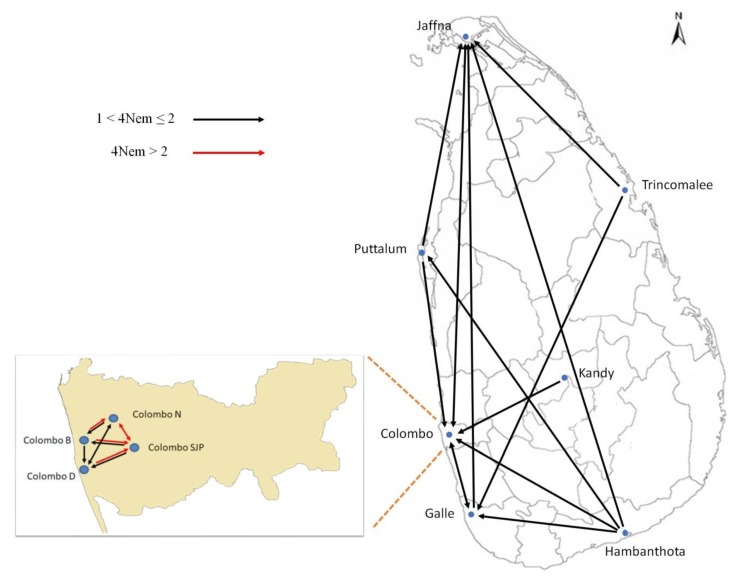
Location of *Ae. aegypti* collection sites in Sri Lanka and estimated patterns of gene flow among these sites. Blue dots indicate the sampling sites and light gray lines indicate the district boundaries on the map. The arrows in the diagram indicate the rate (4N_e_m) and direction of the gene flow among the collection sites as estimated by MIGRATE-n 3.5.1. A significant migration rate is indicated by a black arrow where 4N_e_m > 1. A red arrow indicates 4N_e_m > 2, which is highly significant, and 4N_e_m < 1 is not indicated as it does not imply sufficient migration between the donor and the recipient populations to offset genetic drift. The inset shows the gene flow between the collection sites in Colombo.

The 4NeM values were highly correlated between the two runs (r = 0.753, p < 0.0001), as were values of θ (r = 0.824, p < 0.0034) (Supplementary Figure S3). Mixed gene flow occurred between populations as designated by the arrows in Figure 6, two-headed arrows indicating bi-directional gene flow, one-headed arrows indicating uni-directional gene flow and significant migration rates indicated by black arrows (4Nem > 1) and red arrows (4Nem > 2). All instances where 4Nem < 1, i.e., where sufficient migration between the donor and the recipient populations is not implied to offset genetic drift, is indicated by the absence of arrows (e.g., between Kandy and Trincomalee and between Hambanthota and Trincomalee).

## 4. Discussion

*Aedes aegypti* have a close association with humans and are very well adapted to anthropophilic environments. Passive migration of *Ae. aegypti* to new geographical areas via human activities is a frequently observed scenario. *Aedes aegypti* have been reported in Sri Lanka since the 1930s [25] and this species is now established in almost all districts in the country. 

The present study is the first to analyze multiple *Ae. aegypti* populations within Sri Lanka to investigate gene flow and the population genetic structure. Our results indicate that the populations throughout Sri Lanka are less genetically differentiated with the exception of the Kandy population. PCA and DAPC analysis revealed low genetic structure among the populations studied. The effective migration rate between other cities and Kandy is limited with the exception of moderate gene flow directed toward Colombo, the main commercial capital of Sri Lanka. Although continuous tourist and business activities take place between Kandy and many cities, Kandy reveals an isolated population. Kandy is geographically isolated due to location in the central mountain range in the country. The low temperatures in the mountains may also have played a role in the life cycle of the passively transported mosquito eggs [26,27].

*Aedes aegypti* mosquitoes have a limited active flight range but can move between 10 and 800 m around their larval habitats [28,29]. It has been noted that even though spatial distance affects population structure, human-aided passive dispersal results in migration of mosquitoes to distant locations connected by major roads [30,31,32,33,34,35,36,37]. A study conducted in Pakistan suggested passive dispersal of *Ae. aegypti* mosquitoes in the form of eggs and larvae via tire trade [33]. This study also revealed a low level of isolation by distance and a strong negative correlation between genetic distance and the quality of the road connections [33]. Several other studies have revealed the importance of human-aided passive dispersal for the migration of *Aedes* mosquitoes [37,38]. Weak isolation by distance was noted in Ho Chi Minh City, Phnom Penh and Chiang [30]. A study conducted in Australia, Vietnam and Thailand [36] also suggested that distribution of *Ae. aegypti* populations were connected with human-aided passive migration of the mosquito. 

Our analysis suggests high levels of gene flow into the city of Colombo. Colombo is the main commercial city in Sri Lanka with the main harbor and port situated in the city. Many people move to the city from urban areas daily for their employments. It was also of interest to note that, apart from Colombo, gene flow is also directed toward Galle, which is also another commercial city and a regional port within Sri Lanka with the southern express highway connecting the city of Colombo directly with Galle promoting daily transportation. Similarly, a high level of gene flow is also directed toward Jaffna. With the end of the civil war in 2002, Jaffna has become one of the major tourist attractions. Jaffna and Colombo are connected through direct bus and railway transportation systems. Tourists and commercial supplies are been transported to and from the city of Jaffna via Trincomalee or Puttalum, through which major bus and train routes exist. Major resettlement programs that are being initiated in Jaffna require commercial cargo to be transported to Jaffna. Thus, human-aided passive migration resulting from the high level of commercial traffic between these cities, likely explains the patterns observed in the present study. This conclusion is also strengthened by the weak isolation by distance signal which is likely driven by human-aided movements. 

The lack of migration between other populations suggests that populations within individual cities may be initiated by very few individuals. It has also been reported that *Ae. aegypti* dispersal is restricted by the availability of oviposition sites [39] and the density of the human population [40]. It has been reported that a suitable host availability reduces the dispersal of the mosquitoes [40]. The limited gene flow outside Colombo could be attributed to the high human population density within Colombo and the availability of hosts and breeding sites.

Our analysis with the Garza–Williamson indices suggests genetic drift caused by founder effect. The lowest average Garza Williamson values for the Colombo J and Hambanthota populations suggest recent bottlenecks. Vector control through source reduction and insecticide treatment of breeding sites may also create periodic population bottlenecks leading to genetic drift. *Ae. aegypti* populations in Sri Lanka have been exposed to adulticides and larvicides frequently as dengue eradication campaigns rely on insecticides and removal of breeding sites to lower the disease spread. Populations have therefore experienced intense selection by insecticides that probably resulted in periodic population bottlenecks. A similar scenario has been discussed in Phnom Penh, Cambodia, where treatment of discarded containers with temephos affected the population differentiation [41]. 

The levels of genetic differentiation detected in the current study are low with only one population (Kandy) being genetically differentiated. In comparison, other microsatellite-based studies carried out in the region have recorded much higher levels of genetic differentiation within the study sites [32,33]. Although this could be due to differences in the polymorphism level of the microsatellites used in the current study, the markers appear to have similar diversity when compared with previous studies [32,33]. *Aedes aegypti* females tend to distribute their eggs in multiple oviposition sites, thus a single container may contain a mixture of several female oviposition events, consequently decreasing genetic differentiation [41]. The Kandy population is much more isolated than the rest of the cities due to its geographical location. Analysis with DAPC and *k*-means clustering also revealed an exclusive cluster consisting of Puttalum individuals. This small-scale clustering and the genetic differentiation may be due to the clustering of oviposition sites and hosts. It has been reported that *Ae. aegypti* tends to cluster with a house or a unit of houses [42]. However, the exact spatial barriers that result in the differentiation of Kandy mosquitoes are unknown. 

The geneflow rates and the population genetic structure of *Ae. aegypti* in the present study revealed genetically similar populations with high levels of gene flow. The gene flow was directed toward the major commercial cities. High level of gene flow associated with passive dispersal, aided by human activities and transportation, represents a major threat of dispersal of insecticide-resistant genes to susceptible populations [43,44,45,46,47]. Therefore, these results should be taken in to account in the event of planning control measures for the vector. 

## 5. Conclusions

Our results indicate a passive migration of the *Ae. aegypti* toward the cities of Colombo, Galle and Jaffna via transportation routes. Passive migration of the mosquito to distant areas far from its own flight range may introduce insecticide resistance genes and new pathogens to the more susceptible populations. This could have a serious impact on control programs and the possible threat of the emergence of a disease epidemic. As for future research on the dispersal patterns of the mosquito, examination of *Ae.aegypti* populations from locations in direct trade with Colombo may be informative. Further studies on transportation routes and control strategies could provide better understanding of the population dynamics and the potential spread of *Ae. aegypti* in Sri Lanka thus enabling more informed strategies to control the spread of dengue and other mosquito-borne diseases.

## Figures and Tables

**Table 1:** Analysis of Molecular Variance (AMOVA) of allele frequencies among 10 populations of *Aedes aegypti* in Sri Lanka.

Source of Variation	d.f.	Sum of Squares	Variance Components	F	% Variation
Among cities	6	142.63	0.035	0.048 ***	4.75
Among collections in cities	3	6.46	0.030	0.043 ***	4.08
Within populations	2630	1749.07	0.665	0.088 *	91.17
Total	2639	1898.15	0.729		

Note: * P < 0.05, *** P < 0.0001, d.f.—degrees of freedom.

**Table 2:** Garza Williamson index for the 10 microsatellite loci calculated at each of the 10 populations from *Aedes aegypti* in Sri Lanka.

Locus#	ColomboB	ColomboN	ColomboD	Colombo J	Jaffna	Galle	Hambanthota	Kandy	Trincomalee	Puttalum
AT1	0.750	0.857	0.600	0.800	0.875	1.000	1.000	0.556	0.857	0.667
AG7	0.600	0.571	0.800	0.667	0.750	1.000	0.600	0.800	0.400	0.571
AC1	0.375	0.375	0.375	0.375	0.333	0.208	0.375	0.375	0.375	0.375
AG4	1.000	0.417	1.000	0.333	0.308	0.417	0.364	0.462	0.417	0.115
AG2	0.429	0.500	0.306	0.261	0.600	0.278	0.189	1.000	0.432	0.647
AC2	1.000	1.000	1.000	0.318	1.000	0.417	0.667	1.000	0.049	1.000
AG5	0.625	0.857	0.833	0.833	0.200	0.700	0.571	0.857	0.833	0.833
B07	0.615	0.750	0.778	0.727	0.636	0.875	0.500	0.727	0.500	0.600
H08	1.000	0.600	1.000	0.235	1.000	0.571	0.600	0.714	1.000	1.000
A10	1.000	0.600	1.000	0.800	0.571	0.600	0.500	1.000	0.750	0.500
Mean	0.710	0.653	0.769	**0.535**	0.627	0.607	**0.537**	0.749	**0.561**	0.631
s.d.	0.243	0.206	0.262	0.250	0.285	0.285	0.216	0.227	0.289	0.272

Mean values and standard deviations are displayed per population. The three smallest mean Garza–Williamson indices appear in bold.

## Supplementary Materials

The following are available online at https://www.mdpi.com/2075-4450/11/3/169/s1, Figure S1: Histogram of allele frequencies. Allele frequency distributions at the ten collection sites to discriminate bottlenecked populations from stable populations. The ten intervals along X-axis comprise the bins into which allele frequencies are sorted. Their relative frequencies appear on the Y-axis. Red arrows indicate mode shifts consistent with a past bottleneck. Figure S2: Bayesian information criterion (BIC) for model selection among a finite set of models; the model with the lowest BIC is preferred. Minimum BIC occurred at eight genetic clusters in the dataset. Figure S3: Migrate-n was used to estimate the effective migration rate (4NeM) among the populations. Two replicate maximum likelihood runs were made. Pearson correlation coefficients were calculated between 4NeM values (A) estimated in the first and second replicates to test for the consistency of estimates from the two replicate runs. The same was done with Theta (B). The 4NeMvalues were highly correlated between the two runs (r = 0.753, p < 0.0001), as were values of theta (r = 0.824, p < 0.0034). Supplementary Table S1. Locations, geographic coordinates, and the year of collection of mosquito samples. Supplementary Table S2. Geographic distances (Km) between the study sites in Sri Lanka.
