# Supplementary material for: Gene Flow Patterns among Aedes aegypti (Diptera: Culicidae) Populations in Sri Lanka
Source: Insects. 2020 Mar 6;11(3):169. doi: 10.3390/insects11030169 (PMC7143927; doi:10.3390/insects11030169)
Supplement: Supplementary file 1 [file insects-11-00169-s001.zip › Supplemnetary Figure/Supplementary Table 1.docx]

Supplementary Table 1. Locations, geographic coordinates, and the year of collection of mosquito samples

| Site | Latitude | Longitude | Number of mosquitoes analyzed | Years of collection | Sampling stage |
| --- | --- | --- | --- | --- | --- |
| Colombo - B | 6^0^ 51’ 55” (6.86542) | 79^0^ 52’ 17” (79.8715) | 12 | 2013-2015 | Larvae/adults |
| Colombo- N | 6^0^ 51’ 54” (6.8651) | 79^0^ 54’ 08” (79.9023) | 13 | 2013-2015 | Larvae/adults |
| Colombo - D | 6^0^ 49’ 51.5” (6.8309) | 79^0^ 51’ 46.5” (79.8629) | 10 | 2013-2015 | Larvae/adults |
| Colombo - J | 6^0^ 51’ 16” (6.85445) | 79^0^ 54’ 11” (79.90311) | 9 | 2013-2015 | Larvae/adults |
| Jaffna | 9^0^ 40’ 06” (9.6683) | 80^0^ 0’ 23” (80.0064) | 26 | 2013-2014 | adults |
| Galle | 6^0^ 47’ 00” (6.7833) | 79^0^ 58’ 00” (79.966) | 21 | 2013-2014 | Larvae/adults |
| Hambanthota | 6^0^ 01’ 00” (6.0167) | 80^0^ 46’ 60” (80.7833) | 26 | 2013-2015 | Larvae/adults |
| Puttalum | 8^0^  14’ 00” (8.1667) | 79^0^ 46’ 00” (79.7167) | 30 | 2014-2015 | Larvae |
| Trincomalee | 8^0^ 37’ 00” (8.6167) | 81^0^ 13’ 00” (81.2167) | 28 | 2014-2015 | Larvae |
| Kandy | 7^0^ 17’ 47” (7.2964) | 80^0^ 38’ 06” (80.2167) | 26 | 2013-2015 | Larvae |
