# Supplementary material for: Gene Flow Patterns among Aedes aegypti (Diptera: Culicidae) Populations in Sri Lanka
Source: Insects. 2020 Mar 6;11(3):169. doi: 10.3390/insects11030169 (PMC7143927; doi:10.3390/insects11030169)
Supplement: Supplementary file 1 [file insects-11-00169-s001.zip › Supplemnetary Figure/Supplementary Table 2.docx]

|  | Colombo B | Colombo N | Colombo D | Colombo J | Jaffna | Galle | Hambanthota | Kandy | Trincomalee | Puttalum |
| --- | --- | --- | --- | --- | --- | --- | --- | --- | --- | --- |
| Colombo B | 0 |  |  |  |  |  |  |  |  |  |
| Colombo N | 3.4 | 0 |  |  |  |  |  |  |  |  |
| Colombo D | 3.93 | 5.76 | 0 |  |  |  |  |  |  |  |
| Colombo J | 3.7 | 1.177 | 5.14 | 0 |  |  |  |  |  |  |
| Jaffna | 312 | 311.9 | 315.9 | 313.1 | 0 |  |  |  |  |  |
| Galle | 98.18 | 96.86 | 95.07 | 95.74 | 402.6 | 0 |  |  |  |  |
| Hambanthota | 138 | 135.6 | 136.2 | 134.7 | 415 | 62.32 | 0 |  |  |  |
| Kandy | 96.94 | 94.02 | 99.69 | 94.54 | 272.7 | 145.6 | 143.2 | 0 |  |  |
| Trincomalee | 244.7 | 242.70 | 248.3 | 243.6 | 177 | 305.4 | 293 | 160.2 | 0 |  |
| Puttalum | 152.6 | 152.9 | 156.3 | 154.1 | 161.7 | 247.50 | 270.8 | 141.4 | 165.1 | 0 |

Supplementary Table 2. Geographic distances (Km) between the study sites in Sri Lanka.
