# Supplementary figures and images for: Gene Flow Patterns among Aedes aegypti (Diptera: Culicidae) Populations in Sri Lanka
Source: Insects. 2020 Mar 6;11(3):169. doi: 10.3390/insects11030169 (PMC7143927; doi:10.3390/insects11030169)

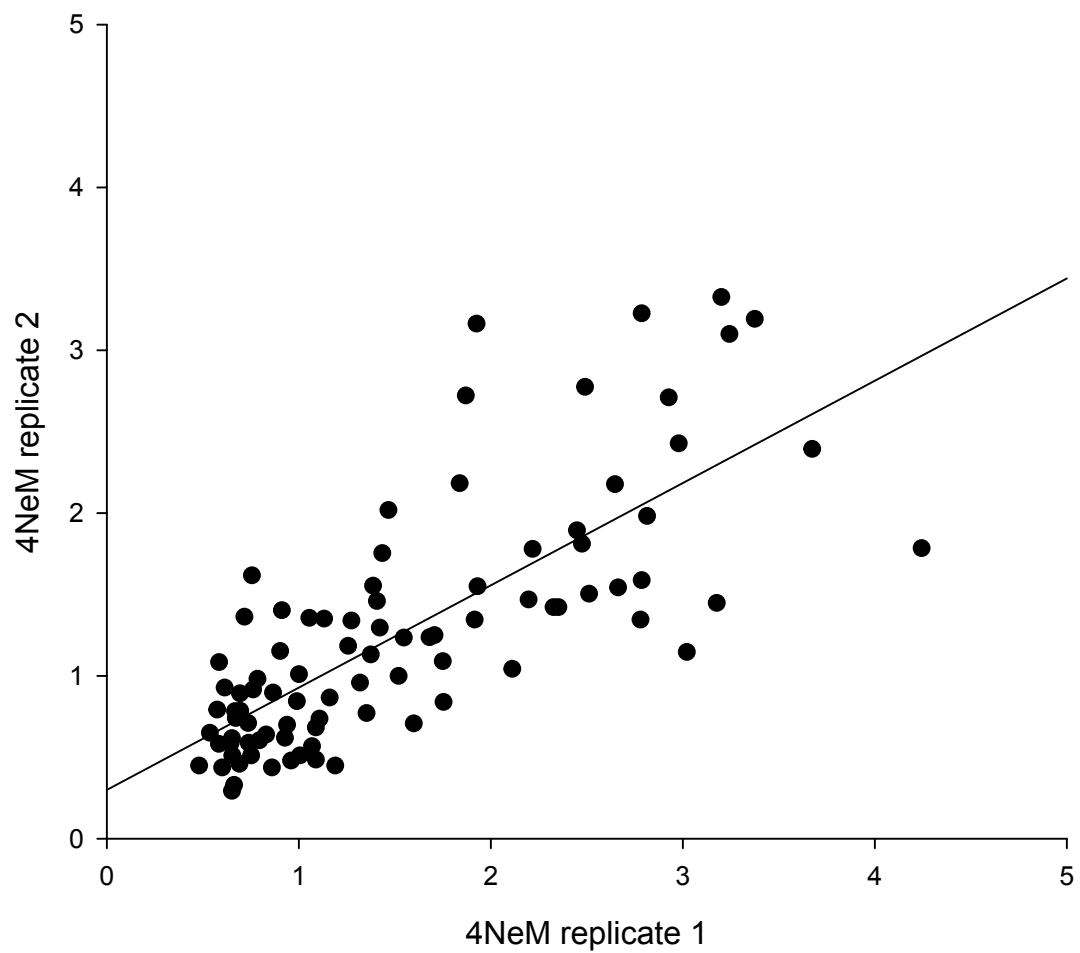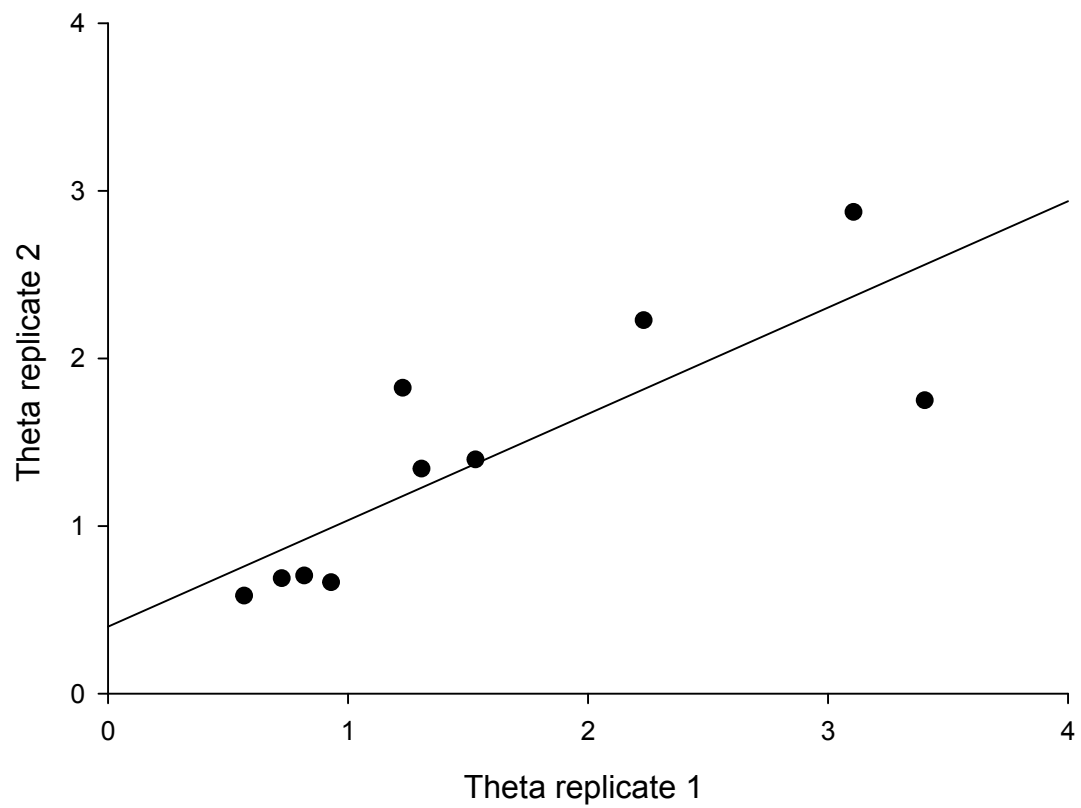

Supplement: Supplementary file 1 [file insects-11-00169-s001.zip › Supplemnetary Figure/Supplement Figure 3.PDF]

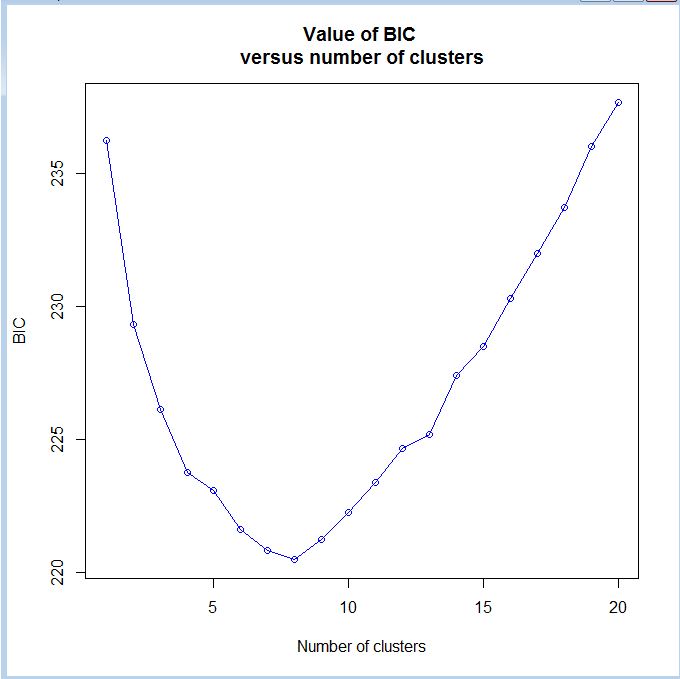

Supplement: Supplementary file 1 [file insects-11-00169-s001.zip › Supplemnetary Figure/Supplementary Figure 2.JPG]

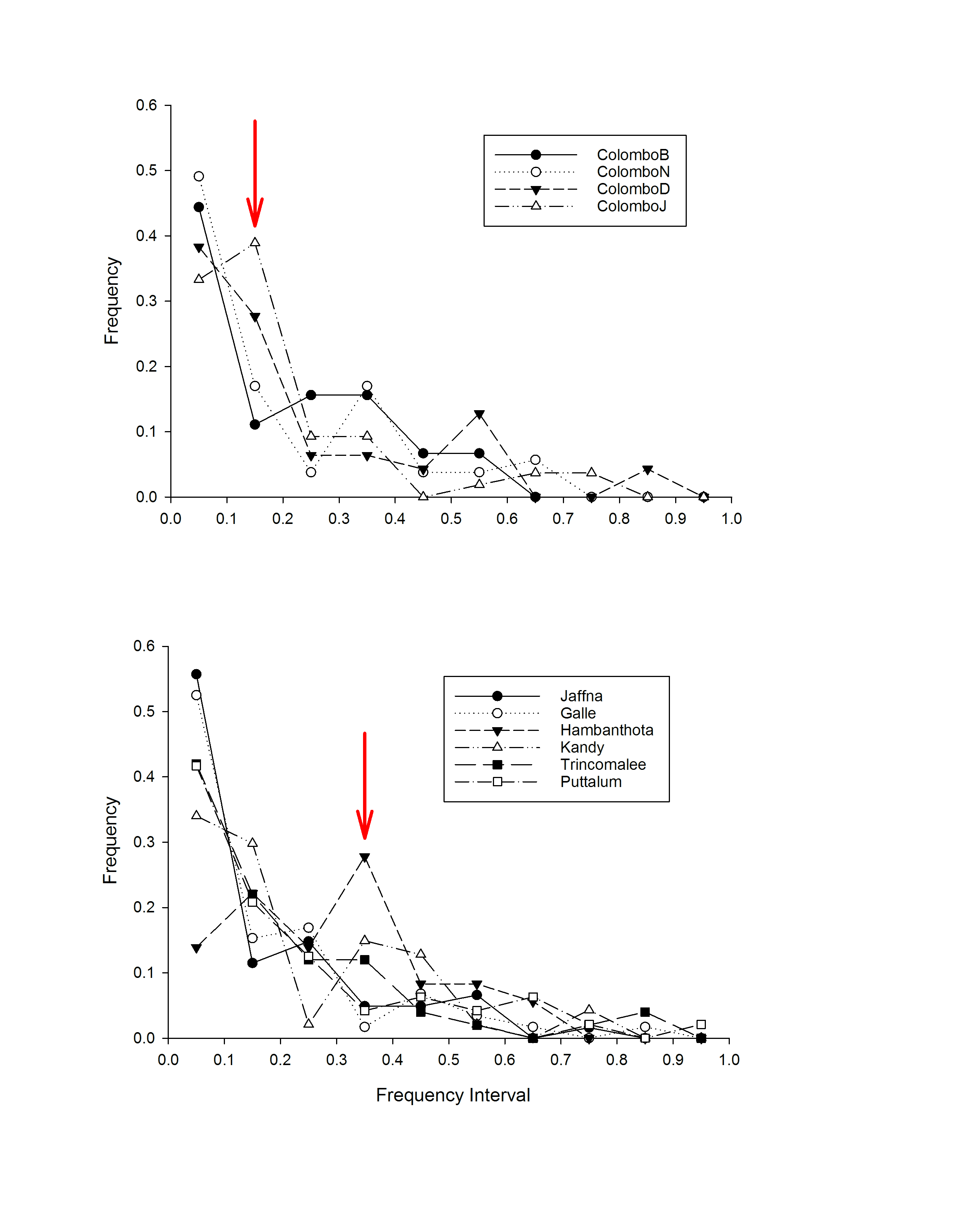

Supplement: Supplementary file 1 [file insects-11-00169-s001.zip › Supplemnetary Figure/Supplemntary FIgure 1.TIF]
